# Phylogeography of the disjunct *Schizolobium parahyba (Fabaceae-Caesalpinioideae)*

**DOI:** 10.1186/1753-6561-5-S7-P12

**Published:** 2011-09-13

**Authors:** Rogerio Margis, Andreia Turchetto-Zolet, Fernanda Cruz, Fabiano Salgueiro, Giovanni Vendramin, Marcelo Simon, Stephen Cavers, Marcia Margis-Pinheiro

**Affiliations:** 1 Universidade Federal do Rio Grande do Sul, Brazil; 2 Universidade Federal do Estado do Rio de Janeiro, Brazil; 3 Plant Genetics Institute – NRC, Brazil; 4 EMBRAPA, Brazil; 5 Center Ecology and Hidrology, Brazil

## 

This work aims to analyze the phylogeography of *Schizolobium parahyba*(Fabaceae), which includes two varieties with a disjunct distribution, from Southern Brazil to Central America. Neotropical rain forests, focus on four largest wet forests: Atlantic forest, Amazonian forests, Andean forest and Central America forest. The genetic diversity and differentiation of populations among *S. parahyba* populations using sequences of three cpDNA regions (*psb*A*-trn*H, *trn*L*-trn*Fand*mat*K) and one nrDNA region (ITS) were analyzed. The presence of the significant phylogeographic structure was inferred by testing if G_ST_ and N_ST_ were significantly different and a spatial analysis of molecular variance was made with both markers.Using cpDNA (*mat*K) sequences of the *S. parahyba* and other Fabaceae species and fossils, we estimated de divergent time for *Schizolobium* clade and using the average ITS substitution rate reported for a range of woody plants, w estimated the divergence time between the two varieties. The high levels of genetic diversity in some populations of *S. parahyba* and two centres of genetic diversity that correlate with the two known varieties: one in the southeast Atlantic forest, and the other in the Amazonian basin*.* In contrast, the populations from Northeast Atlantic forest and Andean-Central America forests showed low level of genetic diversity and divergent haplotypes, probably because the founder effect after dispersion.The results suggest dispersion from southeast Atlantic forest to Amazonian, Andean and Central America forests. We verifya high level of genetic structure, with 68% (nrDNA) and 82% (cpDNA) of the total genetic diversity due to differences among populations. Twenty-one haplotypes were found with cpDNA and four with nrDNA and no haplotypes were shared between varieties.The age for *Schizolobium*clade using *mat*K sequences was estimated ranging from 8.4-23.0 My and the divergence between two varieties using ITS sequence variation was of the 6.5 My. In conclusion, the variation pattern of cpDNA (maternally inherited) and nrDNA (biparentally inherited) markers provides different insights into the phylogeographic structure and gene flow in *S. parahyba.* This comparative analysis of cpDNA and nrDNA markerscan help a deeper understanding of the dynamics responsible for both ancient and more recent events that have shaped the current distribution of genetic variability in Neotropical plants. Theresults are relevant to conservation efforts and ongoing work on the genetics of population divergence and speciation in these Neotropical rainforests. Also, for the long-term conservation of the genetic diversity of *S. parahyba*, including the divergent lineages of the two varieties, it would be important to design strategies that aim to preserve most of its lineages.

